# The Impact of Mouthwash on the Oropharyngeal Microbiota of Men Who Have Sex with Men: a Substudy of the OMEGA Trial

**DOI:** 10.1128/spectrum.01757-21

**Published:** 2022-01-12

**Authors:** Erica L. Plummer, Kate Maddaford, Gerald L. Murray, Christopher K. Fairley, Shivani Pasricha, Andre Mu, Catriona S. Bradshaw, Deborah A. Williamson, Eric P. F. Chow

**Affiliations:** a Centre for Women’s Infectious Diseases Research, The Royal Women’s Hospital, Parkville, Victoria, Australia; b Infection & Immunity Theme, Murdoch Children’s Research Institute, Parkville, Victoria, Australia; c Central Clinical School, Monash University, Melbourne, Victoria, Australia; d Melbourne Sexual Health Centre, Alfred Health, Carlton, Victoria, Australia; e Department of Obstetrics and Gynaecology, The University of Melbourne, Parkville, Victoria, Australia; f Department of Microbiology and Immunology, The Peter Doherty Institute for Infection and Immunity, The University of Melbourne, Melbourne, Victoria, Australia; g Melbourne School of Population and Global Health, The University of Melbourne, Carlton, Victoria, Australia; h Microbiological Diagnostic Unit Public Health Laboratory, The Peter Doherty Institute for Infection and Immunity, The University of Melbourne, Melbourne, Victoria, Australia; i Department of Microbiology, Royal Melbourne Hospital, Melbourne, Victoria, Australia; Emory University

**Keywords:** mouthwash, oropharyngeal gonorrhea, men who have sex with men, *Neisseria gonorrhoeae*, oral microbiome

## Abstract

Mouthwash is a commonly used product and has been proposed as an alternative intervention to prevent gonorrhea transmission. However, the long-term effects of mouthwash on the oral microbiota are largely unknown. We investigated the impact of 12 weeks of daily mouthwash use on the oropharyngeal microbiota in a subset of men who have sex with men who participated in a randomized trial comparing the efficacy of two alcohol-free mouthwashes for the prevention of gonorrhea. We characterized the oropharyngeal microbiota using 16S rRNA gene sequencing of tonsillar fossae samples collected before and after 12 weeks of daily use of Listerine mouthwash or Biotène dry mouth oral rinse. Permutational multivariate analysis of variance (PERMANOVA) was used to assess differences in oropharyngeal microbiota composition following mouthwash use. Differential abundance testing was performed using ALDEx2, with false-discovery rate correction. A total of 306 samples from 153 men were analyzed (Listerine, *n* = 78 and Biotène, *n* = 75). There was no difference in the overall structure of the oropharyngeal microbiota following Listerine or Biotène use (PERMANOVA *P* = 0.413 and *P* = 0.331, respectively). Although no bacterial taxa were significantly differentially abundant following Listerine use, we observed a small but significant decrease in the abundance of both *Streptococcus* and *Leptotrichia* following Biotène use. Overall, our findings suggest that daily use of antiseptic mouthwash has minimal long-term effects on the composition of the oropharyngeal microbiota.

**IMPORTANCE** Given the role of the oral microbiota in human health, it is important to understand if and how external factors influence its composition. Mouthwash use is common in some populations, and the use of antiseptic mouthwash has been proposed as an alternative intervention to prevent gonorrhea transmission. However, the long-term effect of mouthwash use on the oral microbiota composition is largely unknown. We found that daily use of two different commercially available mouthwashes had limited long-term effects on the composition of the oropharyngeal microbiota over a 12-week period. The results from our study and prior studies highlight that different mouthwashes may differentially affect the oral microbiome composition and that further studies are needed to determine if mouthwash use induces short-term changes to the oral microbiota that may have detrimental effects.

## INTRODUCTION

The oral microbial community is highly diverse (1); more than 700 bacterial species have been detected in the mouth using culture-independent methods, and distinct microbial communities can be found on different surfaces and in different locations within the oral cavity (2). Common bacterial genera found in the oral microbiota include *Streptococcus*, *Peptostreptococcus*, *Rothia*, *Actinomyces*, *Moraxella*, *Neisseria*, *Veillonella*, *Prevotella*, *Treponema*, *Leptotrichia*, and *Fusobacterium*, among many others (3). The oral microbiota has an important role in maintaining health, as it can prevent colonization of potentially pathogenic bacteria (3). Given the role of the oral microbiota in human health, it is important to understand if and how external factors influence its composition.

Gonorrhea is a common sexually transmitted infection (STI), with a global incidence of approximately 87 million cases per year (4). Additionally, Neisseria gonorrhoeae displays a high level of genetic plasticity demonstrated by its ability to develop resistance to every class of antimicrobial agent used in current treatment regimens (5, 6). As gonorrhea incidence rises, the likelihood of antimicrobial-resistant N. gonorrhoeae spreading increases; therefore, novel nonantibiotic interventions to prevent the acquisition and control the transmission of infection are urgently required (7, 8).

Men who have sex with men (MSM) are disproportionally affected by N. gonorrhoeae (9, 10), and the oropharynx is postulated to play a key role in gonorrhea transmission in MSM (11); the oropharynx may transmit and acquire N. gonorrhoeae directly, or indirectly via saliva, through sexual contact with a partner’s anus, urethra, and/or oropharynx (11, 12). Because of this, the use of antiseptic mouthwash has been proposed as an intervention to reduce gonorrhea transmission in MSM (7, 13). Previous *in vitro* studies have shown that antiseptic mouthwash can inhibit N. gonorrhoeae growth (14, 15), and a small number of clinical trials have examined the use of mouthwash for the prevention and/or treatment of oropharyngeal gonorrhea (14, 16–19).

Although mouthwash use is common in some populations (20), the long-term effects of mouthwash on the oral microbiota are largely unknown. Therefore, the aim of this substudy was to investigate the effect of 12 weeks of daily mouthwash use on the oropharyngeal microbiota of a subset of men who participated in a randomized trial comparing the use of two mouthwashes for the prevention of oropharyngeal gonorrhea (17). Additionally, we investigated the impact of oropharyngeal gonorrhea infection and smoking on the composition of the oropharyngeal microbiota, as there are currently limited data examining these factors.

## RESULTS

### Study population.

Tonsillar fossae swabs were collected for microbiota analysis from 155 MSM who participated in the Oral Mouthwash use to Eradicate GonorrhoeA (OMEGA) trial (17). Two men did not provide a week 12 sample. As a result, week 0 and week 12 tonsillar fossae samples were available for 153 men, representing 306 samples in total. Seventy-five men were randomized to Biotène and 78 were randomized to Listerine. Baseline characteristics are summarized in Table 1. The median age of participants was 22 years (interquartile range [IQR] = 20 to 24), and 54 men (35%) had oropharyngeal gonorrhea detected by nucleic acid amplification test (NAAT) at baseline.

**TABLE 1:** Baseline characteristics of the study population^*a*^

Characteristic	Value for:		
	Total (*n* = 153)	Biotène (*n* = 75)	Listerine (*n* = 78)
Median age in yrs (IQR)	22 (20–24)	21 (20–24)	23 (21–27)
Country of birth			
Australia	95 (62)	45 (60)	50 (64)
Other^*b*^	58 (38)	30 (40)	28 (36)
HIV status			
Negative	145 (95)	74 (99)	71 (92)
Positive	7 (5)	1 (1)	6 (8)
Current smoker			
No	123 (85)	62 (86)	61 (84)
Yes	22 (15)	10 (14)	12 (16)
Ever used a mouthwash^*c*^			
No	39 (26)	17 (23)	22 (28)
Yes	113 (74)	57 (77)	56 (72)
Current mouthwash use frequency^*c*^			
Never	39 (26)	17 (23)	22 (28)
Every 1–12 mo	44 (29)	24 (32)	20 (26)
Once per wk	30 (20)	15 (20)	15 (19)
Daily	39 (26)	18 (24)	21 (27)
Oropharyngeal gonorrhea at baseline^*d*^			
No	99 (65)	52 (69)	47 (60)
Yes	54 (35)	23 (31)	31 (40)
Oropharyngeal gonorrhea during study period^*d*^			
No	149 (97)	74 (99)	75 (96)
Yes	4 (3)	1 (1)	3 (4)

a Data are presented as *n* (%) or median (IQR); IQR, interquartile range.

b Thirty men reported that their country of birth was from the Western Pacific region, 14 from the European region, 6 from South-East Asian region, 5 from the region of the Americas, 1 from the African region, and 1 from the Eastern Mediterranean region. Country of birth was missing for one man who was born overseas.

c Mouthwash use at baseline not reported by one participant.

d Oropharyngeal gonorrhea diagnosed by NAAT using Aptima Combo 2; Hologic, Marlborough, MA, USA.

### Composition of the oropharyngeal microbiota at baseline.

**FIG 1:**
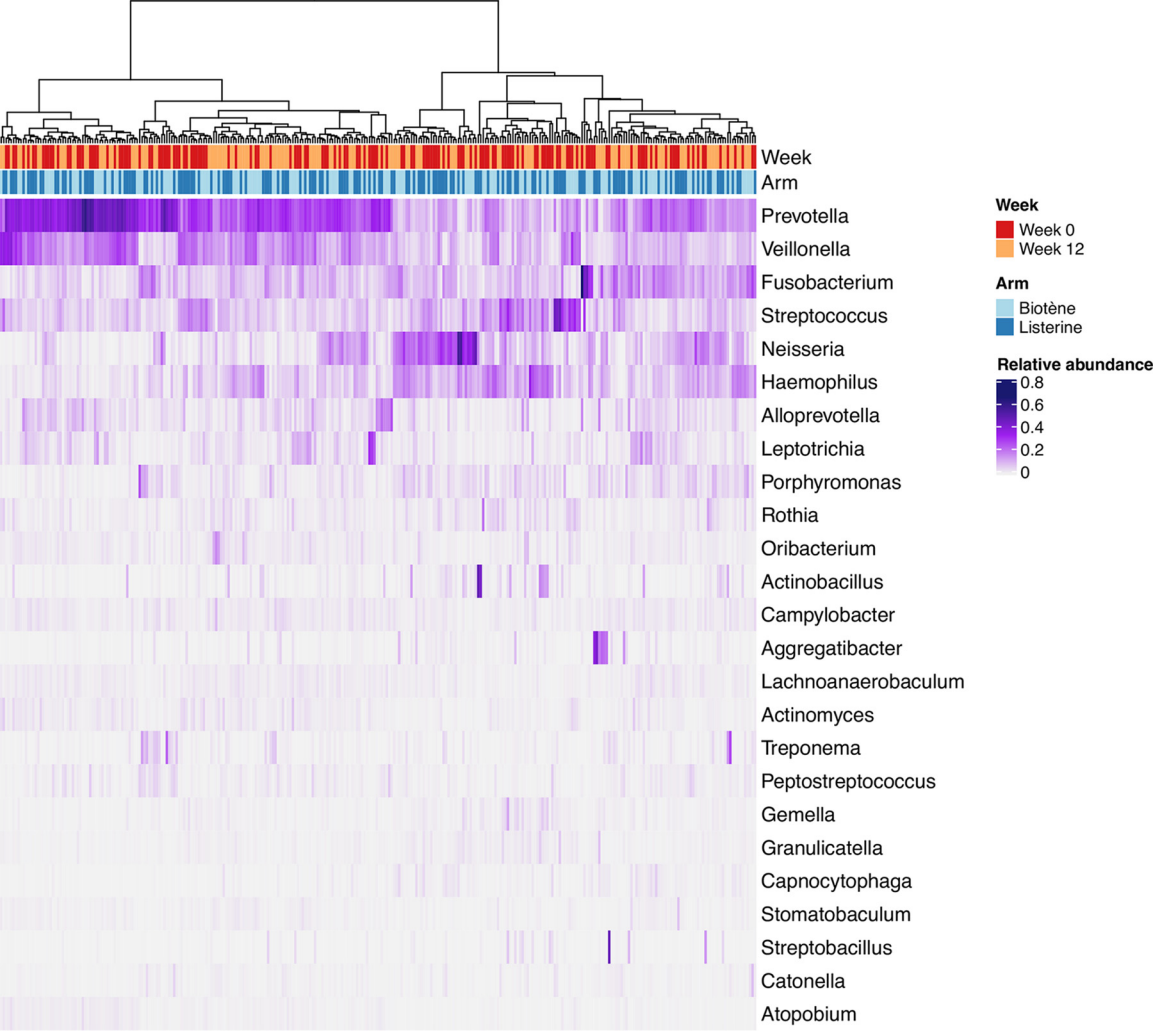
Heatmap of the relative abundance of the 25 most abundant genera detected in all specimens. Each column represents a single tonsillar fossae specimen (*n* = 306 specimens from 153 men), and each row represents a bacterial genus. The dendrogram above the heatmap shows the similarity of microbiota composition between specimens. The metadata above the heatmap indicate the week of collection (i.e., week 0 and week 12) and the randomization group (i.e., Biotène and Listerine).

After quality filtering, the median number of sequencing reads per specimen was 51,425 (IQR = 42,822 to 58,569). The microbiota composition of all specimens is shown in the heatmap in Fig. 1, and there was no obvious clustering of specimens according to randomization arm or week of collection. Bar graphs stratified by randomization group and week of collection are provided in Fig. S1.

Overall, the oropharyngeal microbiota of participants was highly diverse, with a median of 134 amplicon sequence variants (ASVs) detected per sample (IQR = 117 to 150). *Prevotella*, *Veillonella*, *Fusobacterium*, and *Campylobacter* were present in all specimens (*n* = 306), and *Alloprevotella*, *Porphyromonas*, and *Oribacterium* were detected in all but one specimen (*n* = 305). The five most abundant genera were *Prevotella* (median relative abundance 23%, IQR = 14 to 32%), *Veillonella* (11%, IQR = 6 to 18%), *Fusobacterium* (7%, IQR = 4 to 12%), Streptococcus (6%, IQR = 4 to 10%), and *Neisseria* (5%, IQR = 1 to 12%).

### Effect of mouthwash on the composition of the oropharyngeal microbiota.

**FIG 2:**
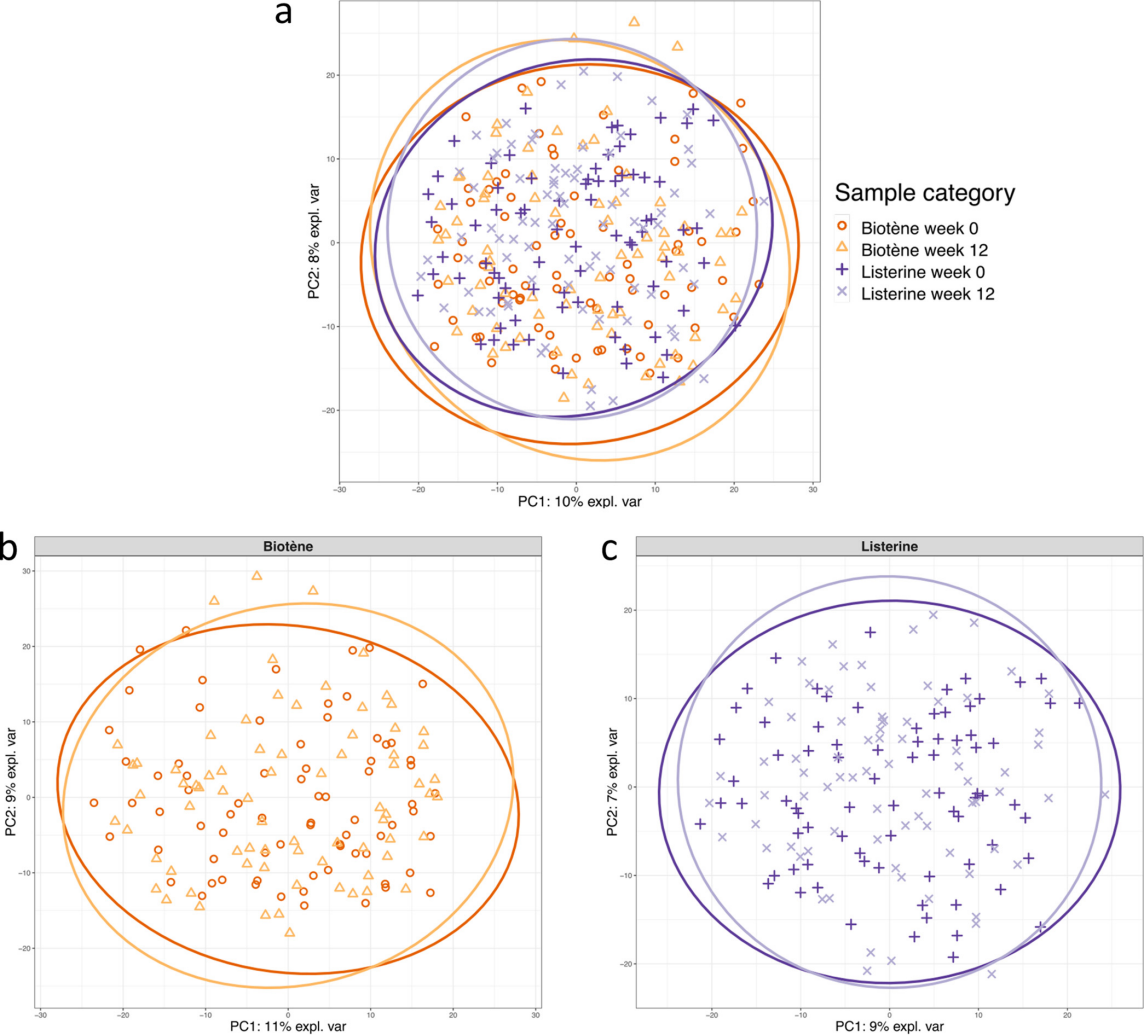
Principal-component analysis (PCA) sample plot of the oropharyngeal microbial communities at week 0 and week 12. (a) The whole study population (*n* = 306 specimens from 153 men). (b) Participants randomized to Biotène (*n* = 150 specimens from 75 men). (c) Participants randomized to Listerine (*n* = 156 specimens from 78 men). Axis labels show the percentage of the total variability in the data set explained by the correspondent axis, and 95% confidence ellipse plots have been included.

Principal-component analysis (PCA) revealed that the composition of the oral microbiota was similar at week 0 and week 12 (Fig. 2). In agreement with the PCA findings, permutational multivariate analysis of variance (PERMANOVA) revealed no differences in oropharyngeal microbial community structure between specimens collected at week 0 and those collected at week 12 (Listerine group: Pseudo-F = 0.99, R^2^ = 0.0064, *P* = 0.413; Biotène group: Pseudo-F = 1.06, R^2^ = 0.0071, *P* = 0.331).

**FIG 3:**
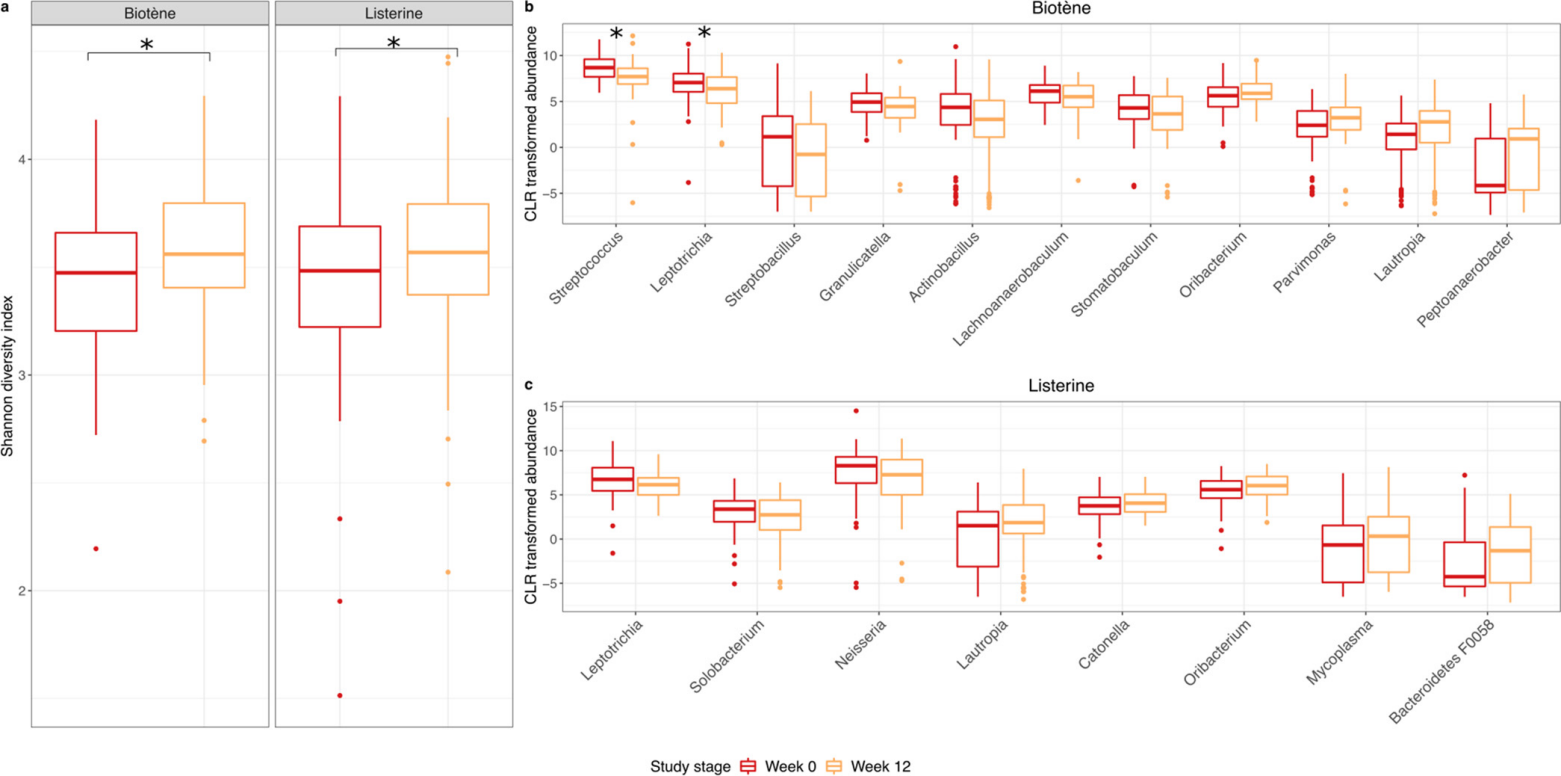
Changes in bacterial diversity and relative abundance following 12 weeks of mouthwash use. (a) Box plots showing the bacterial diversity (measured using the Shannon diversity index) of oropharyngeal samples collected before and after 12 weeks of mouthwash use with either Biotène or Listerine (*, *P* < 0.05). Boxplots showing the center-log ratio (CLR) transformed relative abundance of bacteria that were differentially abundant following 12 weeks of mouthwash use with (b) Biotène and (c) Listerine, as assessed using ALDEx2 (*, FDR-*P* < 0.05; FDR-*P* > 0.05 for all other comparisons; *n* = 306 specimens from 153 men).

There was an increase in the bacterial diversity of the oropharyngeal microbiota at week 12 compared to that at week 0 in both Listerine (median Shannon diversity index of 3.5 [IQR 3.2 to 3.7] at week 0 versus 3.6 [3.4 to 3.8] at week 12, *P* = 0.012) and Biotène participants (median Shannon diversity index of 3.5 [IQR 3.2 to 3.7] at week 0 versus 3.6 [3.4 to 3.8] at week 12, *P* = 0.002) (Fig. 3a); however, the difference in diversity between the two time points was small.

We used ANOVA-like Differential Gene Expression Analysis (ALDEx2) to identify bacterial genera that were differentially abundant between specimens collected at week 0 and those collected at week 12. Seven genera were decreased in abundance at week 12 compared to week 0 in participants using Biotène, and four genera increased in abundance (Fig. 3b). However, following false-discovery rate (FDR) correction, only *Streptococcus* and *Leptotrichia* were significantly decreased after 12 weeks of Biotène use (*Streptococcus* median center-log ratio [CLR] transformed relative abundance at week 0 = 8.68 [IQR 7.68 to 9.59] versus that at week 12 = 7.70 [IQR 6.89 to 8.61], FDR-*P* = 0.004; *Leptotrichia* median CLR transformed relative abundance at week 0 = 7.06 [IQR 6.04 to 8.03] versus that at week 12 = 6.39 [IQR 4.81 to 7.65], FDR-*P* = 0.03). Among participants using Listerine, we identified three genera that were decreased in abundance at week 12 compared to week 0, including *Neisseria*, and five genera that were increased in abundance at week 12 (Fig. 3c). However, following FDR correction, no taxa were significantly differentially abundant after 12 weeks of Listerine use (FDR-*P* of >0.05 for all comparisons). As 39 men reported daily mouthwash use at baseline (Table 1), we conducted a sensitivity analysis where we repeated the above analyses, excluding the 39 men who used mouthwash daily at baseline and one additional participant who did not provide data regarding baseline mouthwash use. The sensitivity analysis yielded similar results for the PERMANOVA analysis (Listerine group: Pseudo-F = 0.98, R^2 ^= 0.0087, *P *= 0.413; Biotène group: Pseudo-F = 0.97, R^2 ^= 0.0087, *P *= 0.432) and for ALDEx2 and diversity analyses (Fig. S2). Similarly, we repeated PERMANOVA analyses including only the 39 men who reported no mouthwash use at baseline, which also yielded similar results (Listerine group: Pseudo-F = 0.72, R^2 ^= 0.0169, *P *= 0.78; Biotène group: Pseudo-F = 0.63, R^2 ^= 0.0192, *P *= 0.915).

As an additional sensitivity analysis, we used PCA and PERMANOVA to compare the overall composition of the oropharyngeal microbiota at baseline between men who reported different mouthwash use frequency. We found no difference in oropharyngeal microbiota composition between men who used mouthwash daily and men who used mouthwash less frequently (Pseudo-F = 0.98, R^2 ^= 0.0065, *P *= 0.413; Fig. S3a). Furthermore, we found no difference in microbiota composition according to mouthwash use frequency when we used four categories of frequency: (i) daily use, (ii) once per week, (iii) every 1 to 12 months, and (iv) never (Pseudo-F = 1.06, R^2^ = 0.0071, *P *= 0.331, Fig. S3b).

Fifty-four men (35%) had oropharyngeal gonorrhea detected by NAAT at baseline (Table 1). To identify any potential impact of gonorrhea infection on the oropharyngeal microbiota, we examined differences in the microbiota composition between men who had oropharyngeal gonorrhea detected by NAAT at baseline (*n* = 54) and men who did not (*n* = 99). PCA and PERMANOVA revealed no differences in the overall composition of the oropharyngeal microbiota of men by infection status (Pseudo-F = 1.56, R2 = 0.01019, *P *= 0.078; Fig. S4a). Using ALDEx2, we identified five genera that had decreased in abundance among men with oropharyngeal gonorrhea detected by NAAT: *Haemophilus*, *Peptostreptococcus*, *Fusobacterium*, *Absconditabacteriales SR1*, and *Bergeyella* (Fig. S4b). However, following FDR correction, no genera were significantly differentially abundant between men who had oropharyngeal gonorrhea detected by NAAT and those who did not (FDR-*P* > 0.05 for all comparisons). Sequencing reads matching N. gonorrhoeae were present in 25/54 men who had oropharyngeal gonorrhea detected by NAAT at week 0 compared to 3/99 men who did not. Additionally, the average relative abundance of N. gonorrhoeae sequencing reads was higher among men who had oropharyngeal gonorrhea detected by NAAT at week 0 than among men who did not (Fig. S5). Only 4 men had oropharyngeal gonorrhea detected by NAAT during the study period; thus, we could not examine differences in the oropharyngeal microbiota composition between these 4 individuals and the 149 men who did not have oropharyngeal gonorrhea detected by NAAT during follow-up.

To identify the impact of smoking on the oral microbiota, we next examined differences in the oropharyngeal microbiota composition between smokers and nonsmokers using specimens collected at week 0. PERMANOVA revealed a small but significant difference in the global oropharyngeal microbiota composition between smokers and nonsmokers (Pseudo-F = 1.8599, R^2 ^= 0.01284, *P *= 0.04; Fig. S6a). Differential abundance analysis using ALDEx2 identified a reduction in *Lautropia* and an increase in both *Treponema* and *Fretibacterium* among smokers compared to those among nonsmokers (Fig. S6b). However, following FDR correction, no genera were significantly differentially abundant by smoking status (FDR-*P* > 0.05 for all comparisons).

To confirm the findings from ALDEx2, we used a second method for differential abundance analysis (analysis of composition of microbiomes; ANCOM [21]). ANCOM results largely agreed with ALDEx2 results (Table S1), with the exception that *Streptobacillus* was found to be significantly decreased following 12 weeks of Biotène use (W-score = 76). *Streptococcus* (W-score = 54) and *Leptotrichia* (W-score = 30) were not significantly differentially abundant by ANCOM. In agreement with ALDEx2, no genera were significantly differentially abundant following Listerine use by ANCOM. Similarly, no taxa were identified as differentially abundant between smokers versus nonsmokers or between men with oropharyngeal gonorrhea detected by NAAT at baseline and those in whom it was not detected.

## DISCUSSION

We investigated the impact of 12 weeks of daily use of two commercially available mouthwashes, Listerine and Biotène, on the oropharyngeal microbiota composition of a subset of men who participated in the OMEGA trial (17). We found no statistically significant difference in the overall composition of the oropharyngeal microbiota after 12 weeks of daily mouthwash use compared to that of the baseline sample, although we did observe a marginal increase in bacterial diversity. While differential abundance analysis revealed no significant changes in the abundance of any genera following Listerine use, we observed a small but significant decrease in the abundance of both *Streptococcus* and *Leptotrichia* following 12 weeks of Biotène use. Overall, our findings suggest that 12 weeks of daily use of Listerine or Biotène has limited long-term effects on the composition of the oropharyngeal microbiota.

Although mouthwash use is common in some populations (20), there are limited published data concerning the impact of mouthwash use on the composition of the oral microbiota, as determined using next-generation sequencing methods. Consistent with our findings, a study of 91 adolescents reported no difference in the overall composition of the supragingival plaque microbiota between individuals using an alcohol-free fluoride-containing mouthwash and individuals using a fluoride-free placebo mouthwash while undergoing fixed orthodontic appliance treatment (22). Another study conducted by the same researchers (23) reported no changes to the dental plaque microbiota composition following 2 weeks of twice-daily use of a mild antimicrobial fluoride-containing mouthwash. However, changes to the overall microbial composition of saliva and tongue samples were reported, including decreased abundance of *Streptococcus* and *Porphyromonas* in saliva samples and decreased abundance of *Veillonella*, *Prevotella*, *Neisseria*, and *Porphyromonas* in tongue samples. In addition, two studies have investigated the effect of 7 days of chlorhexidine-containing mouthwash on the oral microbiota of healthy adults; one investigated the tongue microbiota (24) and the other investigated the salivary microbiota (25). Both studies reported detrimental changes to the oral microbiota following chlorhexidine use, including a reduction in both bacterial diversity and the relative abundance of nitrate-reducing bacteria. A third study investigating mouthwash use for the prevention and treatment of experimental gingivitis found that while chlorhexidine mouthwash induced substantial changes to the subgingival microbiota (including reduced bacterial diversity and a decrease in the abundance of a broad range of bacterial taxa), *N-*acetylcysteine mouthwash was not associated with changes to the subgingival microbiota (26).

Although we observed no changes in the overall structure of the oropharyngeal microbiota following mouthwash use, we observed a small but significant decrease in the relative abundance of both *Streptococcus* and *Leptotrichia* following Biotène use by ALDEx2 and *Streptobacillus* by ANCOM. *Streptococcus* and *Leptotrichia* are common constituents of a healthy oral microbiota (27); however, individual species of both genera have been associated with oral disease (28, 29), and in particular, Streptococcus mutans is considered to be a key pathogen in dental caries (30). *Streptobacillus* has also been detected in the oral microbiota (31) and has been associated with dental caries in children in one study (32). As the V4 region of the 16S rRNA gene has limited ability to distinguish bacteria at the species level (33), particularly *Streptococcus* spp. (34), we could not, therefore, determine if the reduction in *Streptococcus*, *Leptotrichia*, and *Streptobacillus* represents a reduction in commensal species or potentially pathogenic species.

We did not observe significant differences in genera abundance following Listerine use; however, we did observe a nonsignificant change in the abundance of eight genera by ALDEx2. Of particular note, the abundance of *Neisseria* was nonsignificantly decreased in specimens collected after 12 weeks of Listerine use compared to that in specimens collected at week 0 (CLR abundance at week 0 of 8.27 versus that at week 12 of 7.23, *P* = 0.047 and FDR-*P* = 0.35). *Neisseria* species are commonly detected in the oral microbiota of healthy individuals and are often present in high relative abundance (35). There is evidence that commensal *Neisseria* spp. may inhibit pathogenic *Neisseria* spp. through competition of resources or other mechanisms. For example, antagonism between Neisseria elongata and N. gonorrhoeae has been demonstrated *in vitro* and *in vivo* (36). Additionally, Neisseria mucosa isolates have been shown to have antigonococcal activity in delayed antagonism assays, potentially via production of secondary metabolites (37). Furthermore, inoculation of university students with Neisseria lactamica has been shown to reduce nasopharynx carriage of Neisseria meningitidis (38). Therefore, it is possible that a reduction of commensal *Neisseria* in the oral microbiota may increase the risk of N. gonorrhoeae infection. Importantly, however, the OMEGA study found no difference in the incidence of oropharyngeal gonorrhea between men who used Listerine and men who used Biotène, and the overall incidence of oropharyngeal gonorrhea was lower than expected during the 12 weeks of follow-up in both study arms (17). Further studies are needed to understand the relationship between commensal *Neisseria* species and N. gonorrhoeae in the oral environment.

The results from our study and prior studies highlight that different mouthwashes may differentially affect the oral microbiome composition. Factors including the ingredients, alcohol content, and pH of a mouthwash may all influence if and how it modifies the microbiota composition. For example, chlorhexidine is an antiseptic that has broad-spectrum antibacterial activity (39); therefore, one may expect a chlorhexidine mouthwash to have more impact on the oral microbiome compared to milder mouthwashes. Additionally, while exposure to Listerine Zero for 10 s or longer has been shown to inhibit the growth of N. gonorrhoeae *in vitro*, short-term exposure to Biotène (i.e., 5 min or less) has limited activity against N. gonorrhoeae (15, 17). This may indicate that the two mouthwashes have different antimicrobial activity, which could explain, in part, why we observed differences in how the two mouthwashes affected the oropharyngeal microbiota composition. Furthermore, mouthwash exposure is likely to be variable at different anatomical sites in the oral environment, and different sites in the oral cavity have been shown to differ in microbial composition (3, 40). Therefore, the impact of mouthwash at different anatomical sites may also be variable. Overall, further studies are needed to better understand how mouthwash use affects the oral microbiota composition.

In our study, we found no significant difference between the overall oropharyngeal microbiota composition of MSM with and without an oropharyngeal gonorrhea infection. In addition, ALDEx2 analysis revealed no differences in the relative abundance of genera between men with and without an infection. This is perhaps surprising, as N. gonorrhoeae infection has been associated with alterations in the composition of the rectal microbiome (41). Only one other study has described the oral microbiota composition in individuals with oropharyngeal gonorrhea (42). Consistent with our findings, Marangoni et al. (42) reported no differences in the overall structure of the oropharyngeal microbiota in infected versus noninfected men. However, in contrast to our findings, Marangoni et al. (42) found that infected men had an increased abundance of anaerobic bacteria (including *Treponema*, *Parvimonas*, and *Peptococcus*) and a decreased abundance of aerobic bacteria (including *Pseudomonas* and *Escherichia*). Although the differences between the two studies could be a result of population differences, they could also have resulted from differences in statistical method applied. We used ALDEx2 for differential abundance analysis because it accounts for the compositional nature of microbiota data (43) and is generally accepted to be more conservative and have a lower false-positive rate than the standard Wilcoxon test. Additionally, the results from our ALDEx2 analysis were mostly consistent with the findings from a second method for differential abundance analysis (ANCOM).

To our knowledge, Marangoni et al. (42) present the only other analysis of the oral microbiota composition of MSM. We found the composition of the oropharyngeal microbiota of MSM to be very similar to that reported by Marangoni et al. (42), with *Prevotella*, *Veillonella*, *Fusobacterium*, and *Streptococcus* representing the most abundant genera in both studies. While there are no studies to evaluate differences between the oral microbiota composition of MSM and that of men who have sex exclusively with women, similar oral microbiota compositions have been described in other studies of the oral microbiota, including two large studies of Canadian (44) and Japanese (45) adults. In our study, we investigated differences in the oropharyngeal microbiota composition between smokers and nonsmokers at study enrollment. Although we observed a significant difference in the overall microbiota composition by smoking status, smoking explained only 1.2% of the total variance in microbiota composition between individuals, and no genera differed in abundance between smokers and nonsmokers. This was somewhat unexpected because smoking is consistently linked to alterations of the oral microbiota composition (46–49). However, only 15% of men included in our study were smokers, and thus we may have had insufficient power to detect differentially abundant genera. Interestingly, a large study that investigated sources of variability in the oral microbiota composition of 1,049 healthy nonsmoking Canadian adults found that many factors influenced the microbiota composition (44). However, each factor was associated with only small alterations to the microbiota and no single factor accounted for >2% of the total variance between individuals; furthermore, 93% of the variance remained unexplained. This highlights that the oral microbiota is a complex environment that is under multiple external pressures and is likely influenced by several different factors. Importantly, the oral microbiota is considered to be resilient to external influences (50, 51) and its composition has been shown to be very stable over time (52). This resilience and stability may explain, in part, why we observed minimal impact of mouthwash use on the composition of the oropharyngeal microbiota.

There are limitations to this study. First, microbiota samples were collected 12 weeks apart and we advised participants not to use the mouthwash on the day of sample collection. Therefore, we were unable to assess if mouthwash induces transient changes to the oropharyngeal microbiota composition immediately following use. Second, because there were few men included in this substudy who acquired oropharyngeal gonorrhea during follow-up (*n* = 4), and because of the 12-week interval between samples, we were unable to determine if mouthwash use differentially affected the oral microbiota of infected and noninfected men. Future studies incorporating frequent and/or daily sampling following mouthwash use would provide more insight into whether mouthwash use induces immediate and/or short-term changes to the oropharyngeal microbiota and whether and how the oropharyngeal microbiota rebounds following mouthwash use. Third, there are well-known limitations with 16S rRNA gene studies, including limited species-level resolution, and significant biases can be introduced at all steps in microbiota profiling study (53). It is possible that mouthwash use resulted in species (or strain) level changes to the oropharyngeal microbiota, but we were unable to measure these with the methodology we used. Utilizing the full-length 16S rRNA gene or a larger fragment of the 16S rRNA gene would likely provide more insight into species-level changes. Finally, our population comprised MSM who were at high risk of oropharyngeal infection; therefore, our findings may not be generalizable to the general population.

In this study, we report minimal changes to the oropharyngeal microbiota of MSM following 12 weeks of daily use of either Listerine or Biotène, two commercially available mouthwashes. These findings add to our understanding of the impact of mouthwash on the oral microbiota, which is relevant not only for future trials investigating the use of mouthwash for the prevention of oropharyngeal gonorrhea but also for the wider population, given that mouthwash is a commonly used product. Importantly, our findings highlight that further studies with greater resolution and more frequent sampling are needed to determine if mouthwash use induces short-term changes to the oral microbiota.

## MATERIALS AND METHODS

### Participants and specimens.

Participants and specimens used for this study were selected from the OMEGA trial (17). The OMEGA trial was a randomized, double-blind, multicenter, parallel-group trial that compared the efficacy of 12 weeks of daily use of two commercially available alcohol-free mouthwashes for preventing oropharyngeal gonorrhea among MSM who were at high risk for acquiring oropharyngeal gonorrhea (Australian New Zealand Clinical Trials Registry: ACTRN12616000247471). The study population and procedures have been described previously (17). Briefly, MSM aged 16 to 24 who were positive or negative for oropharyngeal gonorrhea in the 30 days prior to enrollment and MSM aged ≥25 years who were positive for oropharyngeal gonorrhea in the 30 days prior to enrollment were eligible for recruitment. At baseline (i.e., week 0), men completed a detailed questionnaire concerning demographic details and sexual practices and were randomized to receive either Listerine Zero (henceforth referred to as Listerine; with *in vitro*-confirmed activity against N. gonorrhoeae) or Biotène Dry Mouth Oral Rinse (henceforth referred to as Biotène); ingredients are provided in supplemental file 1. Men were instructed to rinse and gargle with 20 mL of mouthwash for 60 s at least once a day for 12 weeks.

OMEGA participants recruited at the Melbourne Sexual Health Centre (MSHC) between April 2017 and August 2018 were eligible to participate in this microbiota substudy. Two oropharyngeal swabs (one at the tonsillar fossae and one at the posterior pharyngeal wall) were collected at week 0 by a trained research nurse. Swabs were placed in buffer provided with the Aptima Combo 2 kit (Hologic, Marlborough, MA, USA), and the sample was transferred to a 2 mL tube and stored at −80°C. As part of the OMEGA clinical trial (17), participants also had oropharyngeal swabs collected from the tonsillar fossae and from the posterior pharyngeal wall at weeks 6 and 12. Swabs were placed in Aptima buffer and tested for N. gonorrhoeae by NAAT (Aptima Combo 2; Hologic, Marlborough, MA, USA). The residual Aptima sample was then transferred to a 2 mL tube and stored at −80°C. Tonsillar fossae samples that were collected at weeks 0 and 12 were included in this microbiota substudy.

Alfred Hospital Ethics Committee (HREC/17/Alfred/13) approved this project, and all participants provided written informed consent.

### Laboratory methods.

DNA was extracted from stored tonsillar fossae samples using the QIASymphony PowerFecal Pro kit (Qiagen). A negative (double-distilled water [ddH_2_O]) and a positive control (ZymoBIOMICS Microbial Community standard) were run with each extraction on a 96-well plate. The Earth Microbiome Project (EMP) protocol was used for library preparation (https://earthmicrobiome.org/protocols-and-standards/16s/). Briefly, extracted DNA was used to generate an amplicon-based library using primers that amplify the V4 region of the 16S rRNA gene: 515F (5′-GTGYCAGCMGCCGCGGTAA-3′), 806R (5′-GGACTACNVGGGTWTCTAAT-3′) (54, 55). Libraries (biological samples, as well as positive and negative controls) were sequenced on an Illumina MiSeq instrument (Illumina, San Diego, CA, USA) with a 2 by 150 bp run through Doherty Applied Microbial Genomics at The Peter Doherty Institute for Infection and Immunity, University of Melbourne.

### Sequence and data analysis.

Demultiplexing and trimming of sequencing reads was conducted using the online tool Qiita (https://qiita.ucsd.edu) (56). Reads were demultiplexed using split libraries FASTQ and trimmed to 150 bp (Version QIIMEq2 1.9.1). DADA2 (57) v1.16.0 was used to quality-filter the sequence data, infer amplicon sequence variants (ASVs), and remove chimeras. DADA2 and a DADA2 formatted version of the Silva reference database (v138) (58) were used to assign taxonomy down to the genus level.

Species-level taxonomy for *Neisseria* spp. was performed by a BLAST search against a database of 16S rRNA gene sequences from 53 *Neisseria* strains. ASVs were assigned to species level only if they were an exact match (i.e., had 100% identity) to a type strain. *Neisseria* ASVs that did not have an exact match were assigned to genus level only.

We removed ASVs that were identified as nonbacterial, those that had no phylum assigned, and those that had a total relative abundance of <0.001% and were present in <30% of specimens.

We visually compared the oropharyngeal microbiota composition at weeks 0 and 12 by principal-component analysis of center-log ratio-transformed ASV level sequence data, using mixOmics (v6.12.1) (59). PERMANOVA based on the Bray-Curtis distance was used to test for differences in the overall structure of the oropharyngeal microbiota following 12 weeks of mouthwash use. PERMANOVA was performed using the *adonis* function in vegan (60) with 999 permutations.

Bacterial diversity was calculated on ASV data using the Shannon diversity index using vegan (60) v2.5-7. Changes in bacterial diversity following mouthwash use were assessed using the Wilcoxon signed-rank test.

ASVs with identical taxonomy were merged and a heatmap was generated using ComplexHeatmap v2.5.4 (61). The associated dendrogram was generated with vegan (60) by hierarchical clustering of Bray-Curtis distances with Ward linkage using relative abundance data. Stacked bar plots of the 20 most abundant genera were drawn using ggplot2 (62) and were stratified by week of specimen collection and randomization group.

We used the R package ALDEx2 (43) (ANOVA-like Differential Gene Expression Analysis, v1.20.0) to identify bacterial taxa that were differentially abundant between specimens collected at week 0 and those collected at week 12. Analyses were stratified by randomization group, and we excluded rare taxa (i.e., those present in <10% of samples). ALDEx2 was used to generate 128 Dirichlet Monte Carlo instances using raw sequence counts. Monte Carlo instances were transformed using the center-log ratio transformation and the Wilcoxon signed-rank test, followed by a Benjamini-Hochberg false-discovery rate (FDR) correction. Taxa with an FDR cutoff of <0.05 were considered significantly differentially abundant. To confirm the findings from ALDEx2, we used a second method for differential abundance analysis (Analysis of Composition of microbiomes; ANCOM [21]). For ANCOM analyses, taxa present in <10% of samples were excluded, and a conservative cutoff value of 0.8 was used to identify taxa that were differentially abundant following mouthwash use, using FDR cutoff of <0.05.

PCA, PERMANOVA, and ALDEx2 were also used to investigate differences in the baseline oropharyngeal microbiota composition between individuals with and without specific characteristics/factors. The factors that we investigated were oropharyngeal gonorrhea detection at baseline and smoking.

Sequence and data analysis was performed using R Studio (V1.3.959, Boston, MA, USA) employing R v4.0.2. Sequencing data are publicly available in the NCBI Sequence Read Archive under the project accession number PRJNA759097, and scripts for statistical analysis are available at GitHub (https://github.com/erplummer/omega_microbiota).
