# Supplementary material for: The Impact of Mouthwash on the Oropharyngeal Microbiota of Men Who Have Sex with Men: a Substudy of the OMEGA Trial
Source: Microbiol Spectr. 2022 Jan 12;10(1):e01757-21. doi: 10.1128/spectrum.01757-21 (PMC8754113; doi:10.1128/spectrum.01757-21)
Supplement: SUPPLEMENTAL FILE 1 — Supplemental material. Download SPECTRUM01757-21_Supp_1_seq4.pdf, PDF file, 1.3 MB [file spectrum01757-21_supp_1_seq4.pdf]

Supplementary File 1 – ingredients in Listerine Zero® and Biotène Dry Mouth Oral Rinse®

Listerine Zero®

Ingredients: Water, Sorbitol, Propylene Glycol, Sodium Lauryl Sulfate, Poloxamer 407, Cineole (Eucalyptol), Benzoic Acid, Sodium Benzoate, Methyl Salicylate, Thymol, Sodium Saccharin, Menthol, Sodium Fluoride, Flavour, Sucralose, Green 3 (Cl 42053)

Biotène Dry Mouth Oral Rinse®

Ingredients: Water, Glycerin, Xylitol, Sorbitol, Propylene Glycol, Poloxamer 407, Sodium Benzoate, Hydroxyethylcellulose, Methylparaben, Propylparaben, Flavour, Sodium phosphate, Disodium phosphate

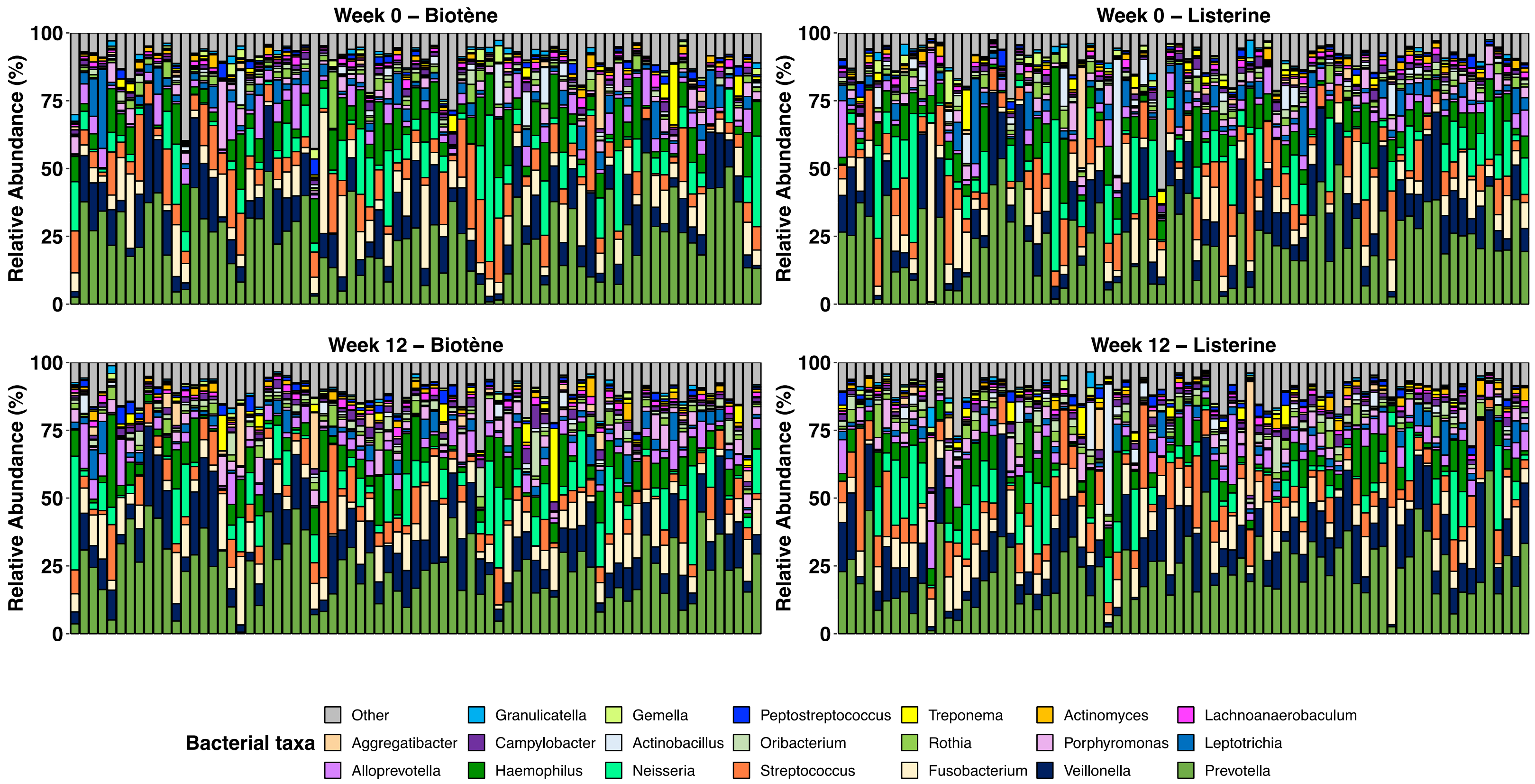

Supplementary Fig. 1 Stacked bar graphs show the relative abundance of the top 20 most abundant bacterial genera in the oropharyngeal microbiota stratified by week of collection and randomisation group. Specimens collected at Week-0 (top row) and Week-12 (bottom row) from the same individuals are displayed in the same order so that an individual's Week-12 specimen appears directly below their Week-0 specimen. 306 specimens from 153 men are included in the figure. All other genera are grouped together in the 'Other' category.

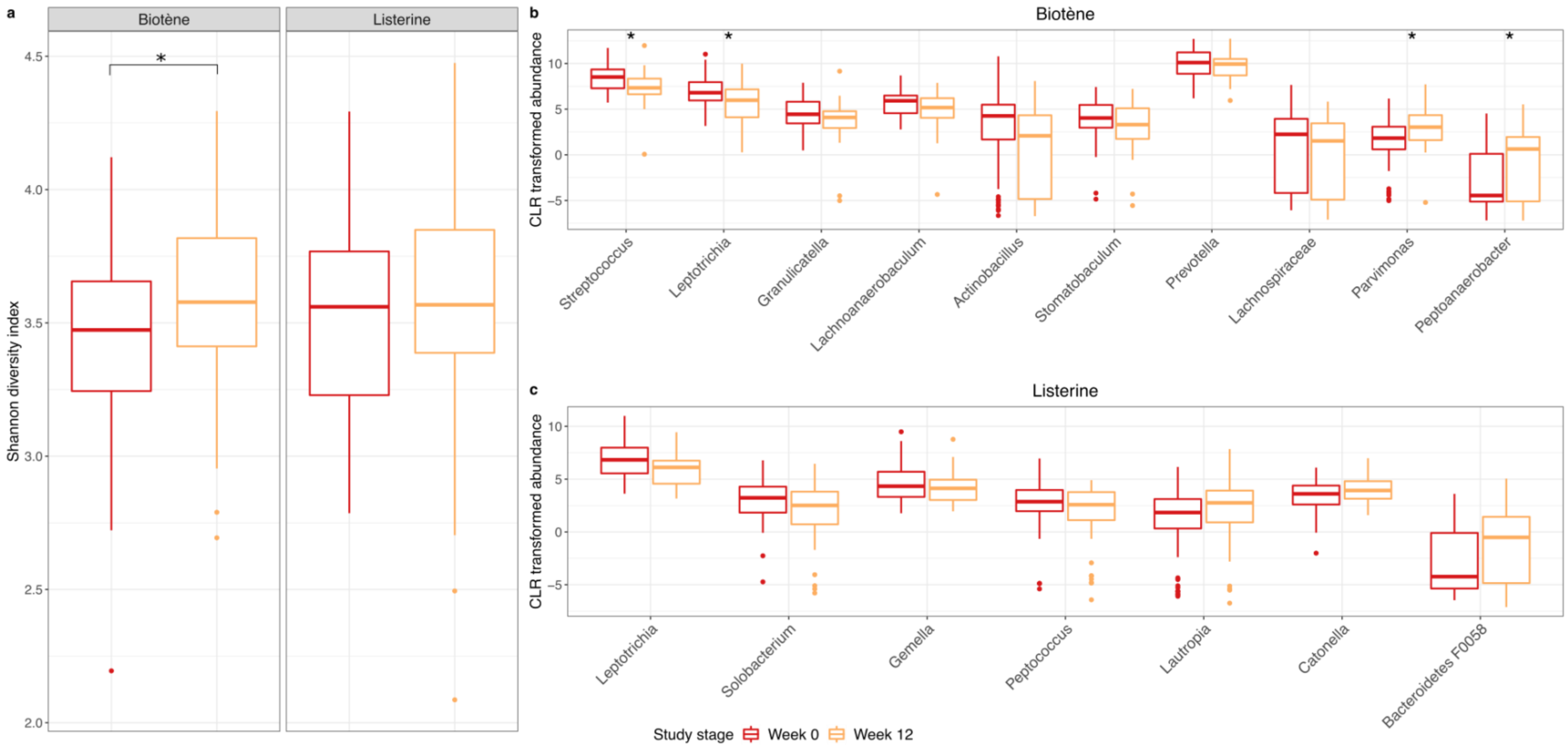

Supplementary Fig. 2 Sensitivity analyses excluding men who reported daily mouthwash use at baseline. (a) Box plots showing the bacterial diversity (measured using the Shannon diversity index) of oropharyngeal samples collected before and after twelve weeks of mouthwash use with either Biotène (median Shannon diversity index= 3.5 [IQR, 3.2-3.7] at week-0 vs 3.6 [3.4-3.8] at week-12, p=0.004; * indicates p<0.05) or Listerine (median Shannon diversity index= 3.6 [IQR, 3.2-3.8] at week-0 vs 3.6 [3.4-3.8] at week-12, p=0.24). Boxplots showing the centred-log ratio (CLR) transformed relative abundance of bacteria that were differentially abundant following 12-weeks of mouthwash use with (**b**) Biotène and (**c**) Listerine, as assessed using ALDEx2 (*FDR-p<0.05; FDR-p>0.05 for all other comparisons)

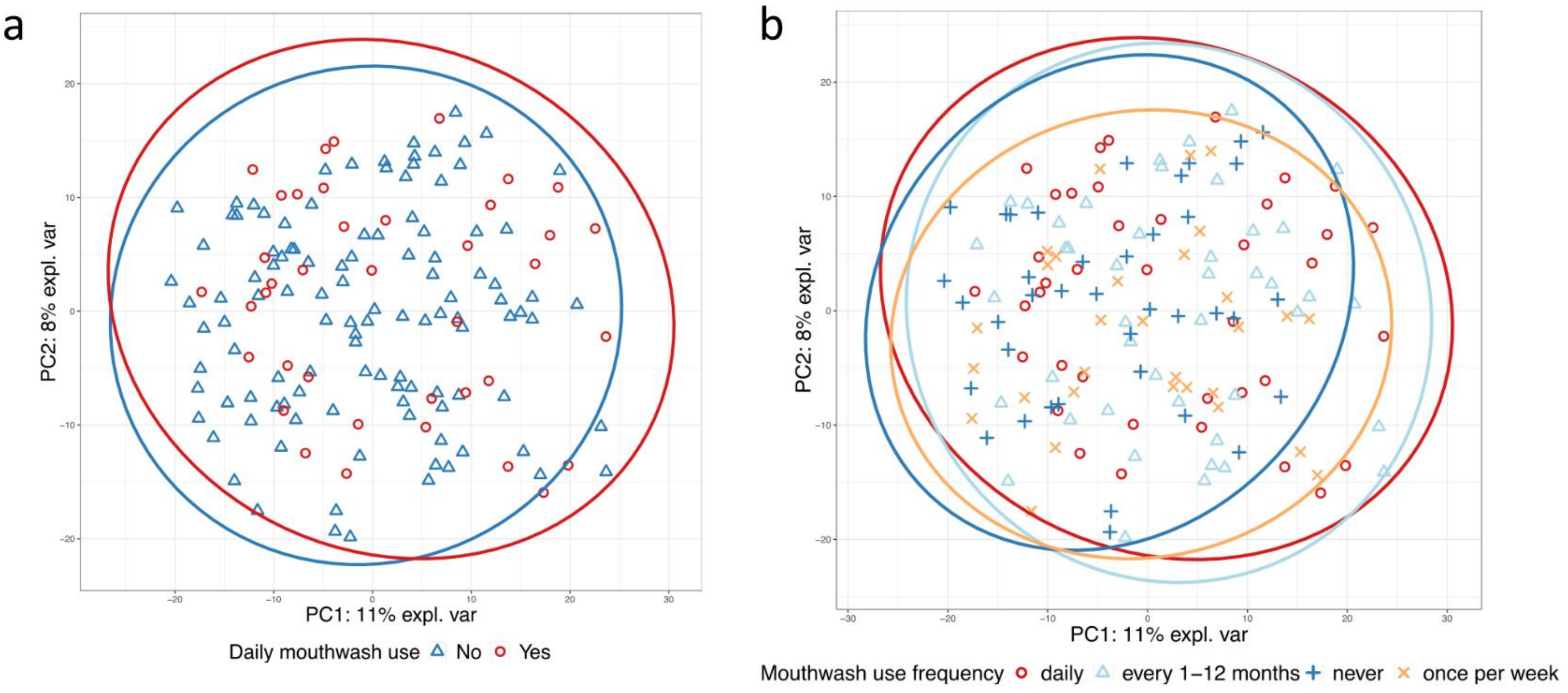

Supplementary Fig. 3 PCA sample plots of the oropharyngeal microbial communities at week-0 in men using mouthwash daily (n=39) and men using mouthwash less than daily (n=139). Axis labels show the percentage of the total variability in the dataset explained by the correspondent axis, and 95% confidence ellipse plots have been included. PERMANOVA revealed no differences in oropharyngeal microbial community structure according to mouthwash use frequency. **(a)** There was no difference in the oropharyngeal microbiota composition of men who used mouthwash daily compared to men who used mouthwash less frequently (Pseudo-F = 0.98, $R^2 = 0.0065$, p = 0.413). **(b)** Similarly, there was no differences according to mouthwash use frequency when analysed as four categories of mouthwash use frequency (Pseudo-F = 1.06, $R^2 = 0.0071$, p = 0.331).

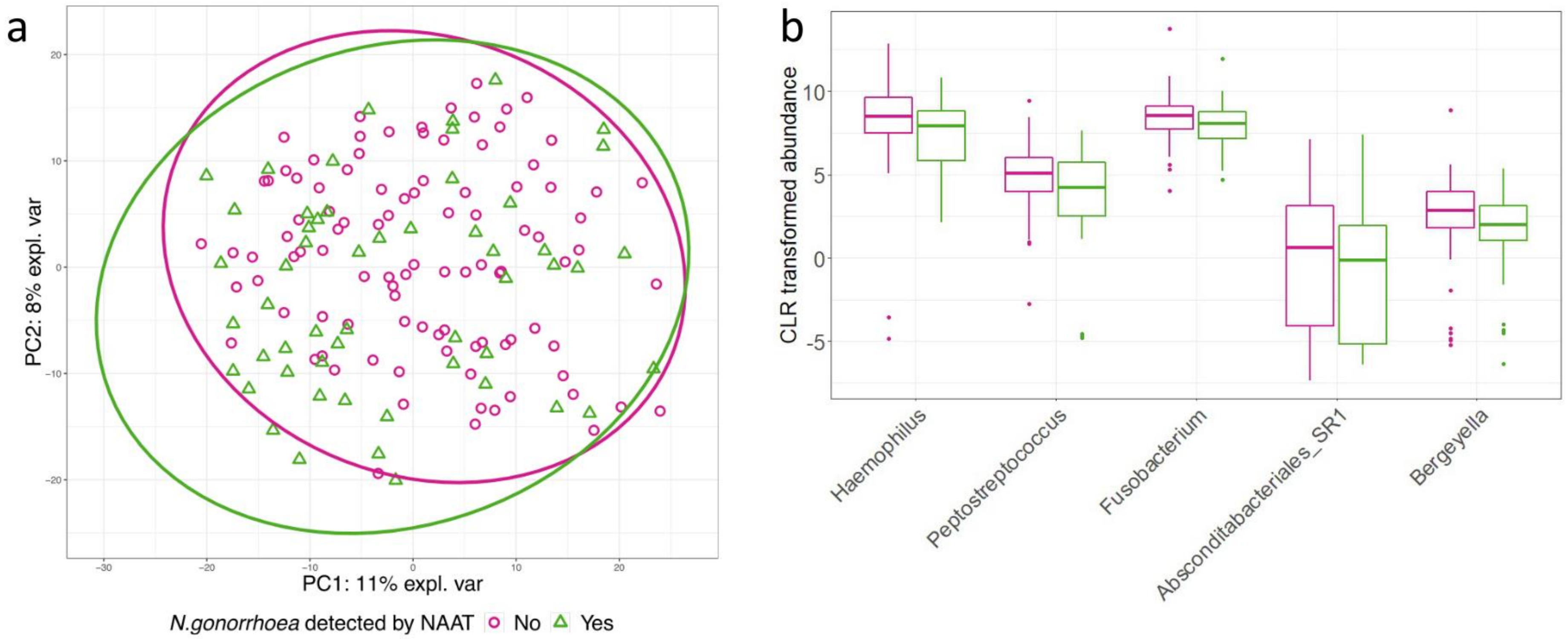

Supplementary Fig. 4 The oropharyngeal microbiota of men with oropharyngeal *N. gonorrhoeae* detected by NAAT at baseline compared to men without oropharyngeal *N. gonorrhoeae*. (a) PCA sample plot of the oropharyngeal microbial communities at week-0 in men with (n=54) and without oropharyngeal *N. gonorrhoeae* detected by NAAT (n=99). Axis labels show the percentage of the total variability in the dataset explained by the correspondent axis, and 95% confidence ellipse plots have been included. PERMANOVA revealed no differences in oropharyngeal microbial community structure according to oropharyngeal gonorrhoea detection status (Pseudo-F = 1.56, R2 = 0.01019, p = 0.078). **(b).** Boxplots showing the centred-log ratio (CLR) transformed relative abundance of bacteria that were differentially abundant (as determined using ALDEx2) between men who had oropharyngeal gonorrhoea detected at week-0 by NAAT and men who did not (FDR-p>0.05 for all comparisons).

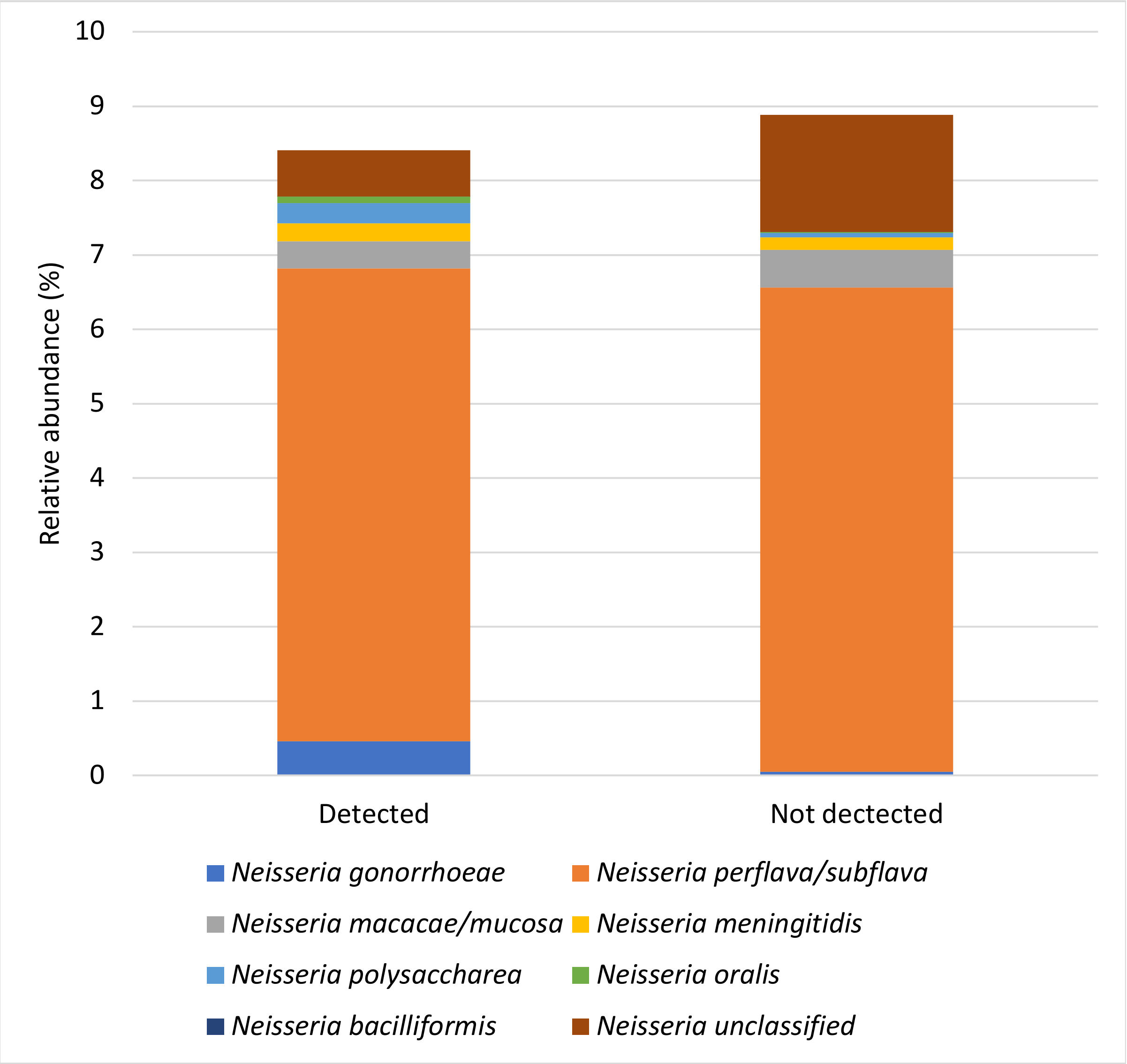

Supplementary Figure 5 – Bar graphs of the average relative abundance of *Neisseria gonorrhoeae* and other *Neisseria* species in men who had oropharyngeal gonorrhoeal detected by NAAT at baseline (n=54), compared to men who did not have oropharyngeal gonorrhoeal detected by NAAT (n=99). *Neisseria* unclassified represents ASVs unable to be assigned to species level.

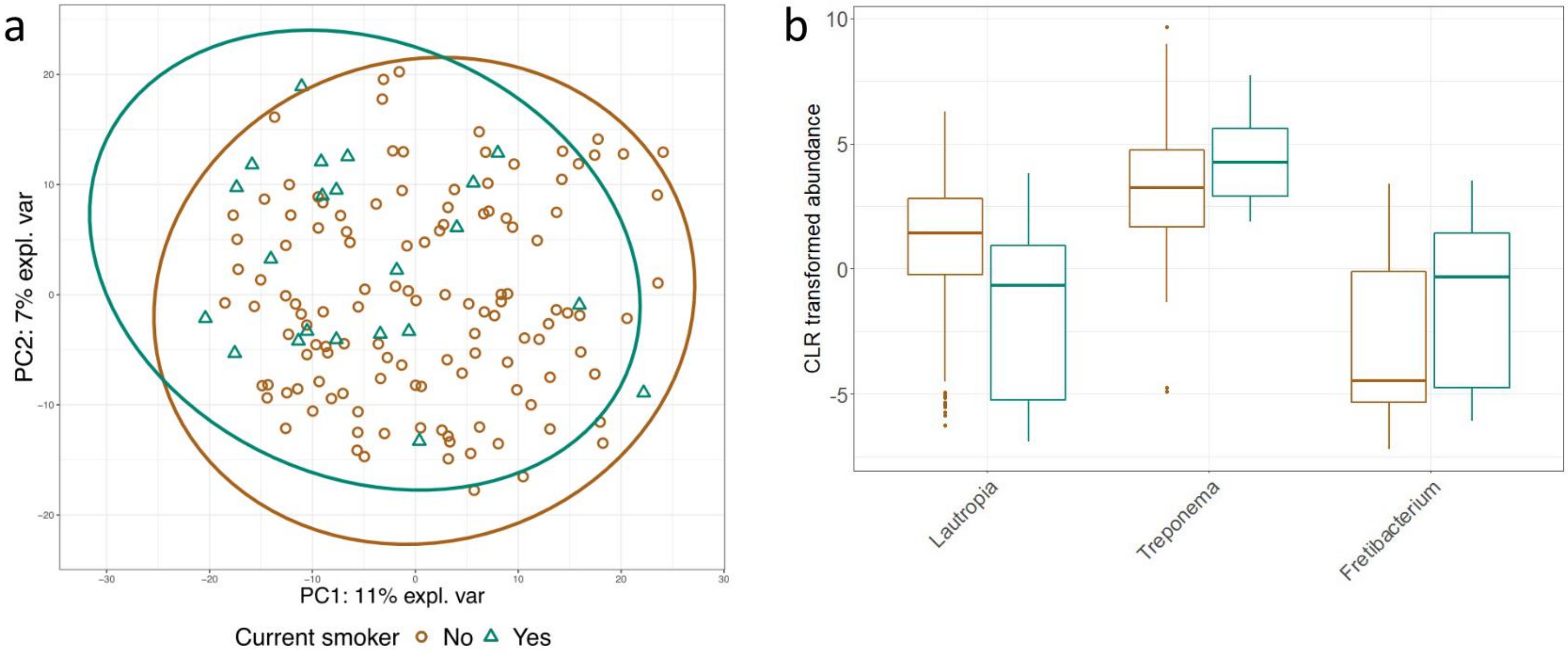

Supplementary Fig. 6 The oropharyngeal microbiota of smokers *vs* non-smokers. (a). PCA sample plot of the oropharyngeal microbial communities at week-0 in smokers (n=22) and non-smokers (n=123). Axis labels show the percentage of the total variability in the dataset explained by the correspondent axis, and 95% confidence ellipse plots have been included. PERMANOVA revealed a small but significant difference between the global oropharyngeal microbiota composition of smokers and non-smokers (Pseudo-F = 1.8599, R2 = 0.01284, p = 0.04). **(b).** Boxplots showing the centred-log ratio (CLR) transformed relative abundance of bacteria that were differentially abundant (as determined using ALDEx2) between smokers and non-smokers at week-0 (FDR-p>0.05 for all comparisons).
